# Interest in patient navigation chatbots: a cross-sectional survey study

**DOI:** 10.3389/fdgth.2026.1785683

**Published:** 2026-09-01

**Authors:** A. Luke MacNeill, Alison Luke, Shelley Doucet

**Affiliations:** 1 Centre for Research in Integrated Care, University of New Brunswick, Saint John, NB, Canada; 2 Faculty of Nursing and Health Sciences, University of New Brunswick, Saint John, NB, Canada

**Keywords:** access, barriers, chatbots, conversational agents, health care, interest, patient navigation, survey

## Abstract

**Introduction:**

Chatbots are accessible and cost-effective tools that may be able to provide navigation support to people who lack access to conventional patient navigation services. To date, little is known about people's interest in using chatbots for patient navigation. It is also unclear what specific types of support people would like to see incorporated into patient navigation chatbots. The purpose of this study was to gain insight into these topics.

**Methods:**

Participants were recruited through the Prolific research platform. They completed a cross-sectional survey that captured information on respondent characteristics, general interest in using a patient navigation chatbot, and interest in various navigation support functions. Data were analyzed using descriptive statistics and ANOVA and hierarchical regression statistical tests.

**Results:**

A total of 276 participants (141 women; *M* age = 38.99 years, *SD* = 12.90) were included in the analysis. In general, participants were moderately interested in using a patient navigation chatbot. They had greater interest in functions related to care coordination, linkage to services and resources, and general education than functions related to needs assessment and treatment support. Interest varied by certain respondent characteristics, particularly demographic and personality factors.

**Discussion:**

The findings from this study will help inform the design and marketing of patient navigation chatbots. The effective implementation of this technology may fill some of the accessibility gaps that characterize conventional patient navigation programs.

## 1 Introduction

Patients and their caregivers often experience barriers to accessing health and social care (1–5). Addressing these barriers is important, as unmet care needs can negatively impact people's health and wellbeing (6–9). Patient navigation is a patient-centered strategy or intervention that aims to remove barriers to care and promote timely access to health and social services (10, 11). It is facilitated by supportive guides known as patient navigators, who help people by assessing their care needs, providing connections to relevant services and resources, and assisting with care coordination, among other activities (12, 13). Research has linked patient navigation to various benefits, including reduced wait times for care, improved patient adherence and health outcomes, and decreased use of hospital and emergency services (11, 14, 15). However, patient navigation programs often rely on short-term private or government funding for operation, which can lead to challenges surrounding program implementation and sustainability (11, 16). As a result, these programs are not available to many patients and caregivers who could benefit from this support.

It may be possible to improve access to navigation support by leveraging certain digital technologies, such as chatbots. Broadly speaking, chatbots are software programs that simulate human conversation through text chats or voice interaction (17, 18). The use of chatbots in the health sector has become more common in recent years, and a growing body of research has demonstrated their utility in this area. For instance, studies have shown that they are an effective means of providing patient education (19, 20), fostering healthy lifestyle habits (21, 22), and addressing mental health concerns (23, 24). These and similar findings suggest that chatbots may be viable tools for delivering navigation support. These tools would be more accessible and cost effective than conventional patient navigation programs, while also offering a more approachable, humanlike interaction experience than alternative software-based solutions (e.g., menu-driven applications). Chatbots would not be a replacement for real patient navigators, as they lack the necessary judgement and skill to handle complex or demanding cases. Regardless, they may be able to provide people with some basic navigation support, which would be particularly beneficial for individuals who would otherwise be left to navigate health and social services on their own.

Although the use of chatbots for patient navigation has considerable potential, there are some important knowledge gaps in this area that require attention. For instance, it is currently unclear whether there is public demand for patient navigation chatbots. Evaluating public interest is key for determining if there is an audience for these tools and the viability of this technology more generally. In addition, it is unclear which types of navigation support people would like to see integrated into patient navigation chatbots. Information on this topic is crucial from a development standpoint, as it would help developers understand how these tools should be designed to enhance user engagement and general appeal. The purpose of the current study was to learn more about these topics. We developed and administered an online survey to examine people's interest in using a patient navigation chatbot, as well as their interest in various support functions. In addition to assessing broad interest across our sample, we evaluated whether there were individual differences in participants' interest levels. It is possible that interest varies by certain respondent characteristics (e.g., demographics), and learning more about these differences would allow developers to tailor their design and marketing strategies for specific populations.

## 2 Methods

### 2.1 Design

We conducted an exploratory, cross-sectional survey study with an online sample of participants from Canada and the United States. The study survey assessed general interest in using a patient navigation chatbot, as well as interest in various navigation support functions. The survey also captured information on several respondent characteristics, which was used to assess variations in participant interest.

Respondent characteristics consisted of demographic, personality, and health factors that have been linked to variables closely related to interest (e.g., attitudes, utilization) in past digital health research. Demographic factors included age, gender, income, and education (25–27); personality factors included conscientiousness and neuroticism (28–30); and health factors included health status and digital health literacy (26, 31, 32). Two additional respondent characteristics were assessed for exploratory purposes. Country of residence was included as a demographic factor as we thought that structural differences in the Canadian and American health care systems could influence people's navigation needs and preferences. Access to a primary care provider was included as a health factor to account for the possibility that people without a primary care provider are more likely than others to need guidance or assistance with respect to navigation.

This study was approved by the research ethics board at the University of New Brunswick and is reported in accordance with STROBE cross-sectional reporting guidelines (33).

### 2.2 Participants

Participants were recruited through the online research platform Prolific (Prolific, London, UK). A power analysis conducted using G*Power version 3.1.9.7 (Heinrich-Heine-Universität, Düsseldorf, Germany) indicated that 123 participants would be required for the study (.80 power, *α* level = .05, *f*^2^ = .15). However, we sought a higher number of participants (*N* > 250) to provide a more robust analysis. To qualify for the study, individuals had to be residents of Canada or the United States aged 19 years or older. They also had to have a successful completion rate of 97% or above on their Prolific tasks. Filters were used to ensure a similar number of Canadian and American participants were recruited, as well as a similar number of men and women. Recruitment occurred in July and August 2023. Participants were provided with $3.00 (American) for their participation.

### 2.3 Measures

An initial questionnaire asked demographic questions about participant age, gender, annual household income, education, and country of residence. Canadian incomes were converted to American funds following data collection. The demographic questions were accompanied by a health-related question asking whether participants had a primary care provider, such as a family doctor, a general practitioner, or a nurse practitioner who provides primary care. Additional measures are described in detail below.

#### 2.3.1 Personality factors

Personality factors were measured with items from the Mini-International Personality Item Pool Scales (Mini-IPIP; 34). The two personality factors of interest in this study were conscientiousness, which indicates a characteristic tendency to be organized, responsible, and planful; and neuroticism, which indicates a characteristic tendency to be anxious, nervous, and emotionally unstable (35). Conscientiousness and neuroticism were assessed with four items apiece. Each item asked participants how well a personality-related quality or behavior describes them. Items were rated on a scale ranging from 1 (*very inaccurate*) to 5 (*very accurate*). Responses to certain items were reverse scored, such that higher ratings reflect higher levels of a personality factor. Responses to the items were averaged within each personality factor to obtain composite scores for each participant. The conscientiousness items demonstrated acceptable internal consistency (Cronbach's *α* = .75), whereas the neuroticism items demonstrated good internal consistency (Cronbach's *α* = .84).

#### 2.3.2 Health status

Health status was measured with the Short-Form Health Survey (SF-12; 36). Each of the 12 items on this measure asked about a specific aspect of a person's health and wellbeing. Items were rated on a scale with two to six response options, depending on the item. Two summary scores were generated using a scoring algorithm: one score for physical health and another score for mental health (37). In both instances, higher scores indicate better health. A score of 50 indicates average health.

#### 2.3.3 Digital health literacy

Digital health literacy was measured with the Digital Health Literacy Instrument (DHLI; 38). Digital health literacy refers to the ability to find, evaluate, and apply online health information and use digital health applications (32, 38). We considered it a health factor for this study, although it also taps into people's technological skills. Each of the 21 items on the DHLI asked about the difficulty or frequency of performing tasks related to digital health use. Items were rated on a scale ranging from 1 (*very easy* or *never*) to 4 (*very difficult* or *often*). All responses were reverse scored, such that higher scores reflect higher levels of digital health literacy. Responses to the items were averaged to obtain a composite score for each participant. The DHLI items demonstrated good internal consistency (Cronbach's *α* = .88).

#### 2.3.4 Chatbot interest

Chatbot interest was evaluated with six items created for this study. The first item assessed participants' general interest in using a patient navigation chatbot. The remaining five items assessed their interest in specific navigation support functions: (1) *performing needs assessments* (e.g., identifying health and social care needs); (2) *assisting with care coordination* (e.g., helping with appointment scheduling); (3) *linking to services and resources* (e.g., linking to social and financial services and resources); (4) *providing treatment support* (e.g., discussing medical results and treatment plans); and (5) *providing general education* (e.g., teaching how to navigate the health care system). These items were based on tasks and activities performed by real patient navigators (11–13, 39), with a focus on behaviors that could be feasibly performed by a chatbot. Items were rated on a scale ranging from 1 (*not at all interested*) to 5 (*extremely interested*). The full measure is presented in Supplementary Material 1.

### 2.4 Procedure

Participants were redirected from Prolific to the Qualtrics online survey platform (Qualtrics, Provo, USA), which hosted the study materials. They began by completing an informed consent form and the demographic, personality, and health measures. Next, they were provided with basic information on chatbots (e.g., input and output modalities) and shown example screenshots of chatbots that are used for health care (40, 41). The purpose of this material was to ensure that all participants had a sufficient understanding of this technology before continuing with the study. This material addressed chatbots in a general sense, without specifying an underlying system architecture (rule-based, artificial intelligence, etc.) or introducing overly technical details. After reviewing this material, participants filled out the item assessing their general interest in using a patient navigation chatbot, as well as the items assessing their interest in the navigation support functions.

### 2.5 Data analysis

Data were entered into SPSS version 29 (IBM Corp., Armonk, USA) for statistical analysis. Continuous and scale variables were summarized using means and standard deviations, and categorical variables were summarized using frequencies. Note that a median split was performed on income due to heavy skew and potential bimodality in the distribution (peaks in the $45–50,000 range and the $65–70,000 range; *N* = 22 each). The education categories were grouped into post-secondary degree/diploma vs. no post-secondary degree/diploma due to imbalance across the original categories (skewed toward higher education); there was a strong Spearman correlation between the dichotomized variable and the original ordering (*ρ* = .82). Participants' interest in the five navigation support functions was compared using a repeated measures analysis of variance (Greenhouse-Geisser correction *ε* = .88); the omnibus test was followed up with *post hoc* Bonferroni tests.

Individual differences in participants' interest ratings were assessed using a series of six hierarchical multiple regressions. The outcome variable for the first regression was general interest in using a patient navigation chatbot, and the outcome variables for the five remaining regressions were interest in the five navigation support functions. The predictor variables for the regressions were the same in each analysis. They were entered in three successive steps according to presumed temporal or conceptual priority (42, 43). Demographic factors were included at Step 1 as relatively stable, foundational variables. Personality factors, which represent enduring patterns of thoughts, feelings, and behaviors (44, 45), were added at Step 2 to see if they explained additional variance beyond demographic factors. Health factors, which include more modifiable, downstream characteristics that are affected by both demographics and personality (46–49), were added at Step 3 to see if they explained additional variance beyond demographic and personality variables. No adjustments were made to the alpha level (.05) for the regressions due to the exploratory nature of the analyses and the moderate to strong correlations between the outcome variables (50, 51).

## 3 Results

### 3.1 Participant details

A total of 303 individuals participated in the study. Participants were excluded from the analysis for completing the survey in an improbable time frame, i.e., <5 min (*N* = 7); for failing quality check questions embedded in the survey (*N* = 4); for missing large amounts of data (*N* = 2); for being flagged by Qualtrics's fraud detection tools (*N* = 1); and for demonstrating monotonic responding (*N* = 1). In addition, some participants were excluded as statistical outliers (*N* = 10) or categorical (e.g., gender) outliers (*N* = 2). Following these exclusions, 276 participants remained for the analysis. A summary of their demographic characteristics is presented in Table 1, along with descriptive statistics for the personality and health variables.

**Table 1:** Descriptive statistics for demographic, personality, and health variables.

Variable	Statistic
Age in years, *M* (*SD*)	38.99 (12.90)
Gender, *N*	
Man	135
Woman	141
Household income, *N*	
Under $62,000 (U.S.)	138
$62,000 (U.S.) and over	138
Education, *N*	
No post-secondary degree/diploma	78
Post-secondary degree/diploma	198
Country, *N*	
Canada	139
United States	137
Mini-IPIP Conscientiousness, *M* (*SD*)	3.63 (0.85)
Mini-IPIP Neuroticism, *M* (*SD*)	2.84 (1.04)
SF-12 Physical health, *M* (*SD*)	50.35 (8.21)
SF-12 Mental health, *M* (*SD*)	42.94 (12.24)
DHLI Digital health literacy, *M* (*SD*)	3.39 (0.34)
Primary care provider, *N*	
Yes	215
No	61

The median household income ($62,000) was included with the higher income group to best balance the *N* between the two groups.

Mini-IPIP, Mini-International Personality Item Pool Scales; SF-12, Short-Form Health Survey; DHLI, Digital Health Literacy Instrument.

### 3.2 Overall interest

Across the sample, participants reported moderate interest in using a patient navigation chatbot (*M* = 3.36, *SD* = 1.12). They were moderately to very interested in the five navigation support functions, with interest ratings differing significantly across these functions, *F*(3.53, 971.73) = 24.86, *p* < .001, *η*_p_^2^ = .08. More specifically, participants reported greater interest in care coordination (*M* = 3.77, *SD* = 1.06), linkage to services and resources (*M* = 3.85, *SD* = 0.95), and general education (*M* = 3.75, *SD* = 1.05) functions than they did in needs assessment (*M* = 3.49, *SD* = 1.09) and treatment support (*M* = 3.32, *SD* = 1.24) functions (all pairwise *p* < .001). No significant differences were found among the three functions of higher interest (all *p* > .729). Among the two functions of lower interest, participants were more interested in a needs assessment function than they were in a treatment support function (*p* = .029).

### 3.3 Individual differences in interest

The regression results indicated that there were individual differences in the outcome variables. These results are reported in detail in Table 2. Although demographic factors did not explain variation in participants' interest in using a patient navigation chatbot, they did account for interest in several navigation support functions. More specifically, American residents were more interested in both a needs assessment function and a care coordination function than Canadian residents; people with a lower household income were more interested in a services and resources function than those with a higher household income; and people who were younger and had lower education levels were more interested in a treatment support function than those who were older and had higher education levels. Personality factors accounted for additional variance in two outcome variables beyond demographic factors. This result was largely due to conscientiousness: people with higher levels of conscientiousness reported greater interest in using a patient navigation chatbot than those with lower levels of conscientiousness, and they also reported greater interest in a treatment support function. Health factors did not account for additional variance in the outcome variables beyond demographic and personality factors. More information on the regression model results is reported in Supplementary Material 2. Bivariate correlations are reported in Supplementary Material 3.

**Table 2:** Summary of regression results.

Variable	General interest		Needs assessment		Care coordination		Services and resources		Treatment support		General education	
	Δ*R*^2^	β	Δ*R*^2^	β	Δ*R*^2^	β	Δ*R*^2^	β	Δ*R*^2^	β	Δ*R*^2^	β
Step 1	.03		.07**		.05**		.06**		.07**		.03	
Age		0.00		−0.06		−0.11		−0.04		−0.16**		−0.07
Gender		0.05		0.05		0.06		0.09		−0.04		0.02
Household income		−0.06		−0.09		−0.11		−0.19**		−0.01		−0.09
Education		−0.11		−0.10		−0.04		−0.06		−0.14*		−0.08
Country		0.05		0.19**		0.14*		−0.02		0.11		0.05
Step 2	.03*		.01		.02		.00		.05***		.01	
Mini-IPIP Conscientiousness		0.20**		0.12		0.14		0.00		0.26***		0.12
Mini-IPIP Neuroticism		0.03		0.02		0.10		0.00		0.07		0.01
Step 3	.03		.01		.03		.03		.01		.01	
SF-12 Physical health		−0.12		−0.10		−0.18		−0.15		−0.11		−0.05
SF-12 Mental health		−0.20		−0.09		−0.11		−0.17		−0.07		−0.10
DHLI Digital health literacy		0.00		−0.01		0.03		0.08		−0.01		−0.01
Primary care provider		0.04		0.01		−0.01		0.05		0.04		0.01

Coefficients are not interpreted unless they contribute to a significant Δ*R*^2^. More information on the regression model results is reported in Supplementary Material 2.

Mini-IPIP, Mini-International Personality Item Pool Scales; SF-12, Short-Form Health Survey; DHLI, Digital Health Literacy Instrument.

* *p* < .05.

** *p* < .01.

*** *p* < .001.

## 4 Discussion

### 4.1 Interpretation of results

Participants in this study tended to report moderate interest in using a patient navigation chatbot. The average interest rating across the sample was just above the midpoint of the rating scale, which was more indicative of cautious interest than strong enthusiasm. These findings are consistent with previous studies showing mixed or slightly positive attitudes toward the use of chatbots for health care (52, 53). Research suggests that people's cautiousness or hesitancy over health chatbots can be attributed to several factors, such as concerns about information accuracy and control over personal health data (53). Patient navigation chatbots would likely pose less risk than other types of health chatbots, as their primary focus would be on improving access to care rather than providing care directly. Regardless, the perception of risk may persist and could be affecting individuals' interest levels.

Although participants reported some interest in each of the assessed support functions, they seemed to be most interested in functions related to care coordination, linkage to services and resources, and general education. Notably, the needs assessment and treatment support functions received the least amount of interest, even though the provision of needs assessments and treatment support are among the most common activities performed by real patient navigators (13). Lower interest in these functions could be due to a number of factors. For instance, people may lack confidence in a chatbot's abilities to perform these specific tasks, which would typically require a high level of training and expertise; they may fail to recognize the importance of these tasks within a navigation context; or they may simply have less interest in these types of support more generally.

We identified several individual differences in the study results. For instance, general interest in using a patient navigation chatbot was greater among participants with higher levels of conscientiousness. Conscientious individuals are organized, responsible, and planful by nature (35), and research indicates that they engage in greater self-management of health (e.g., more preventive health behaviors) than people with lower conscientiousness (54, 55). This focus on health management may stimulate interest in accessible and user-friendly tools for maintaining and improving their health. Consistent with this idea, studies have found that people who are higher in conscientiousness are more likely than others to use and have positive perceptions of digital health (28, 29). The results of the current study suggest that their receptivity to digital health interventions extends to patient navigation chatbots. Conscientious individuals' focus on health management might also explain their apparent interest in having a treatment support function incorporated into this technology; this function would be a valuable resource for health maintenance and improvement.

In terms of demographic differences, Americans reported greater interest in the needs assessment and care coordination functions than Canadians. The American health care system is more fragmented than the Canadian system from a structural standpoint (56, 57) and tends to show poorer performance on measures of administrative efficiency and access to care (58). These issues may help explain why Americans are drawn to chatbot functions that are intended to help with the planning and coordination of care. Additionally, lower-income participants reported greater interest in a function that links people to services and resources than higher-income participants. It makes intuitive sense that people with comparatively few socioeconomic resources would be interested in a function that can facilitate connections to financial, logistical (e.g., transportation), or similar resources; however, it is worth noting that these same people experience barriers to accessing and using digital technologies (59), which might constrain their ability to benefit from digital health applications. Finally, participants who were younger and had lesser education reported greater interest in a treatment support function than their older and more educated counterparts. These individuals may have seen a treatment support function as a way to fill the health-related knowledge gaps that accompany a lack of life experience or formal learning.

### 4.2 Practical implications

The findings from this study will prove useful for the design and marketing of patient navigation chatbots. Although many people seem to lack strong interest in using chatbots for patient navigation, developers would likely find a receptive audience in individuals with higher levels of conscientiousness. To broaden the appeal of this technology, developers should incorporate high-interest functions such as assisting with care coordination, linking to services and resources, and providing general education on navigation and related topics. At the same time, it may be worthwhile to emphasize specific functions when targeting certain populations. For instance, a chatbot that links people to services and resources would have particular appeal for lower income individuals, whereas a chatbot that provides treatment support would be more likely to attract youth, people with lesser education, and conscientious individuals. Similarly, a chatbot that can help with the management of care (e.g., conducting needs assessments and coordinating care) may appeal to Americans and other individuals who face particularly complex or fragmented health care systems. Developers may be able to leverage this collective information to increase the adoption and use of their chatbots, which in turn could improve navigation-related outcomes for patients and their caregivers.

### 4.3 Limitations

There were several limitations to this study. First, participants were recruited through the online research platform Prolific. The fact that they were recruited via online methods suggests that they may be more inclined toward digital health technologies than the general public and that our results lack a certain degree of generalizability. Our participants also had demographic differences compared to the general population (e.g., higher levels of education), which might have affected the findings. Second, hierarchical regression results are sensitive to the order in which predictor variables are entered into the analysis. Alternative orderings based on other underlying rationales may yield different interpretations or insights. Third, the research design used in this study is not able to establish causal relationships among the study variables, which has implications for the regression analysis. Other research designs should be used to confirm the influence of our predictor variables on people's interest. Similarly, our results cannot establish the reasons or explanations for any significant effects, which should be explored using other designs. The differences related to country of residence, in particular, should be explored further; although we proposed that structural differences in the Canadian and American health care systems could account for these results, broader social or cultural factors may have contributed. Finally, it should be noted that participants' interest in using a patient navigation chatbot may not be an accurate indicator of their future use of these tools. Barriers such as a lack of access to the necessary technologies and poor literacy skills may result in a discrepancy between individuals' interest and their actual usage.

## 5 Conclusion

Chatbots may be able to help fill the accessibility gaps that characterize conventional patient navigation programs. However, their impact in this area will depend on their effective implementation. Developers will be able to draw on the results of this study when developing and marketing their patient navigation chatbots to help ensure that this technology is delivered in a manner that fully realizes its potential.

## Data Availability

The raw data supporting the conclusions of this article will be made available by the authors, without undue reservation.
