# Supplementary material for: Interest in patient navigation chatbots: a cross-sectional survey study
Source: Front Digit Health. 2026 Sep 1;8:1785683. doi: 10.3389/fdgth.2026.1785683 (PMC13574941; doi:10.3389/fdgth.2026.1785683)
Supplement: Supplementary file 1 [file Supplementaryfile1.docx]

Supplementary Material 1

# Patient Navigation Chatbot Questionnaire

Imagine a chatbot that is designed to help patients navigate the health care system. The goal of this chatbot would be to improve access to health services, address barriers to care, and promote greater coordination and integration of care. Please rate your interest in using this type of chatbot on a scale of 1 (*Not at all interested*) to 5 (*Extremely interested*).

| Not at all  interested  1 | Slightly  interested  2 | Moderately interested  3 | Very  interested  4 | Extremely  interested  5 |
| --- | --- | --- | --- | --- |

| Which of the following functions would you like to see included in a navigation chatbot? Please rate your interest on a scale of 1 (*Not at all interested*) to 5 (*Extremely interested*). | | | | | | | |
| --- | --- | --- | --- | --- | --- | --- | --- |
|  |  | **Not at all interested** | | | **Extremely interested** | | |
| The ability to identify patient needs and barriers (e.g., identify health and social care needs; identify barriers to accessing health and social services) |  | 1 | 2 | 3 | | 4 | 5 |
| The ability to assist with care coordination (e.g., help patients with appointment scheduling; prepare patients for medical appointments). |  | 1 | 2 | 3 | | 4 | 5 |
| The ability to link patients to services and resources (e.g., link patients to social and financial services and resources; link patients to transportation services and resources). |  | 1 | 2 | 3 | | 4 | 5 |
| The ability to provide treatment support (e.g., discuss patients’ medical results and treatment plans; provide social and emotional support). |  | 1 | 2 | 3 | | 4 | 5 |
| The ability to provide general education (e.g., provide general health information; teach patients how to navigate the health care system). |  | 1 | 2 | 3 | | 4 | 5 |
