# Supplementary material for: Interest in patient navigation chatbots: a cross-sectional survey study
Source: Front Digit Health. 2026 Sep 1;8:1785683. doi: 10.3389/fdgth.2026.1785683 (PMC13574941; doi:10.3389/fdgth.2026.1785683)
Supplement: Supplementary file 2 [file Supplementaryfile2.docx]

Supplementary Material 2

# Regression Model Results

**General Interest**

Step 1: *R*^2^ = .03, *F* (5, 270) = 1.43, *p* = .213

Step 2: Δ*R*^2^ = .03, Δ*F* (2, 268) = 4.61, *p* = .011

Step 3: Δ*R*^2^ = .03, Δ*F* (4, 264) = 1.82, *p* = .126

Full regression model: *R*^2^ = .08, *F* (11, 264) = 2.19, *p* = .016

**Needs Assessment**

Step 1: *R*^2^ = .07, *F* (5, 270) = 4.04, *p* = .001

Step 2: Δ*R*^2^ = .01, Δ*F* (2, 268) = 1.67, *p* = .191

Step 3: Δ*R*^2^ = .01, Δ*F* (4, 264) = 0.79, *p* = .532

Full regression model: *R*^2^ = .09, *F* (11, 264) = 2.43, *p* = .007

**Care Coordination**

Step 1: *R*^2^ = .05, *F* (5, 270) = 3.14, *p* = .009

Step 2: Δ*R*^2^ = .02, Δ*F* (2, 268) = 2.23, *p* = .109

Step 3: Δ*R*^2^ = .03, Δ*F* (4, 264) = 2.03, *p* = .090

Full regression model: *R*^2^ = .10, *F* (11, 264) = 2.61, *p* = .004

**Services and Resources**

Step 1: *R*^2^ = .06, *F* (5, 270) = 3.37, *p* = .006

Step 2: Δ*R*^2^ = .00, Δ*F* (2, 268) = 0.00, *p* = .998

Step 3: Δ*R*^2^ = .03, Δ*F* (4, 264) = 2.15, *p* = .075

Full regression model: *R*^2^ = .09, *F* (11, 264) = 2.33, *p* = .009

**Treatment Support**

Step 1: *R*^2^ = .07, *F* (5, 270) = 3.81, *p* = .002

Step 2: Δ*R*^2^ = .05, Δ*F* (2, 268) = 7.84, *p* < .001

Step 3: Δ*R*^2^ = .01, Δ*F* (4, 264) = 1.01, *p* = .404

Full regression model: *R*^2^ = .13, *F* (11, 264) = 3.61, *p* < .001

**General Education**

Step 1: *R*^2^ = .03, *F* (5, 270) = 1.59, *p* = .162

Step 2: Δ*R*^2^ = .01, Δ*F* (2, 268) = 1.88, *p* = .155

Step 3: Δ*R*^2^ = .01, Δ*F* (4, 264) = 0.35, *p* = .842

Full regression model: *R*^2^ = .05, *F* (11, 264) = 1.19, *p* = .295
