# Supplementary material for: Interest in patient navigation chatbots: a cross-sectional survey study
Source: Front Digit Health. 2026 Sep 1;8:1785683. doi: 10.3389/fdgth.2026.1785683 (PMC13574941; doi:10.3389/fdgth.2026.1785683)
Supplement: Supplementary file 3 [file Supplementaryfile3.docx]

Supplementary Material 3

# Correlations Between Study Variables

(Table continued on next page)

|  | 1. | 2. | 3. | 4. | 5. | 6. | 7. | 8. |
| --- | --- | --- | --- | --- | --- | --- | --- | --- |
| 1. Age | -- |  |  |  |  |  |  |  |
| 2. Gender | -.01 | -- |  |  |  |  |  |  |
| 3. Income | .05 | -.02 | --  *** |  |  |  |  |  |
| 4. Education | .05 | .11 | .34 | -- |  |  |  |  |
| 5. Country | .10  *** | .01 | -.07  * | -.07  * | -- |  |  |  |
| 6. Mini-IPIP Cons. | .22  *** | .03  *** | .13  ** | .14  * | .09 | --  ***  ** |  |  |
| 7. Mini-IPIP Neur. | -.23  *** | .20 | -.18  *** | -.15  ** | -.17 | -.50 | -- |  |
| 8. SF-12 Physical | -.25  *** | -.04 | .21  ** | .16  ** | -.07  ** | .02  *** | -.06  *** | -- |
| 9. SF-12 Mental | .23 | -.12 | .17  * | .20 | .17 | .48  *** | -.73  *** | -.07  ** |
| 10. DHLI Dig. Health Lit. | .10 | -.03  *** | .14  * | .11 | .11 | .25 | -.34 | .17 |
| 11. Primary Care Provider | -.11 | -.21 | -.13 | -.01  * | .05 | -.08  ** | -.06 | .05 |
| 12. General Interest | .00 | .04 | -.10  * | -.12  * | .07  ** | .15 | -.03 | -.12 |
| 13. Needs Assessment | -.06 | .04 | -.14  * | -.14 | .20  * | .08 | -.01 | -.11  * |
| 14. Care Coordination | -.10 | .07 | -.14  *** | -.08  * | .14 | .06 | .07 | -.15  * |
| 15. Services and Resources | -.05  ** | .09 | -.22 | -.12  ** | -.01 | -.04  ** | .08 | -.14 |
| 16. Treatment Support | -.16 | -.06 | -.08  * | -.17 | .11 | .16 | -.02 | -.08 |
| 17. General Education | -.08 | .02 | -.12 | -.11 | .05 | .08 | -.01 | -.05 |

*Note.* Mini-IPIP = Mini-International Personality Item Pool Scales; SF-12 = Short-Form Health Survey; DHLI = Digital Health Literacy Instrument. * = *p* < .05. ** = *p* < .01. *** = *p* < .001.

|  | 9. | 10. | 11. | 12. | 13. | 14. | 15. | 16. | 17. |
| --- | --- | --- | --- | --- | --- | --- | --- | --- | --- |
| 1. Age |  |  |  |  |  |  |  |  |  |
| 2. Gender |  |  |  |  |  |  |  |  |  |
| 3. Income |  |  |  |  |  |  |  |  |  |
| 4. Education |  |  |  |  |  |  |  |  |  |
| 5. Country |  |  |  |  |  |  |  |  |  |
| 6. Mini-IPIP Cons. |  |  |  |  |  |  |  |  |  |
| 7. Mini-IPIP Neur. |  |  |  |  |  |  |  |  |  |
| 8. SF-12 Physical |  |  |  |  |  |  |  |  |  |
| 9. SF-12 Mental | --  *** |  |  |  |  |  |  |  |  |
| 10. DHLI Dig. Health Lit. | .26 | -- |  |  |  |  |  |  |  |
| 11. Primary Care Provider | .05 | -.02 | -- |  |  |  |  |  |  |
| 12. General Interest | -.04 | .00 | .01 | --  *** |  |  |  |  |  |
| 13. Needs Assessment | -.02 | -.02 | .02 | .62  *** | --  *** |  |  |  |  |
| 14. Care Coordination | -.07  * | -.01 | -.02 | .50  *** | .57  *** | --  *** |  |  |  |
| 15. Services and Resources | -.13 | .00 | .04 | .54  *** | .56  *** | .59  *** | --  *** |  |  |
| 16. Treatment Support | .00 | -.01 | .05 | .66  *** | .66  *** | .46  *** | .41  *** | --  *** |  |
| 17. General Education | -.03 | -.02 | .01 | .53 | .60 | .45 | .56 | .52 | -- |

*Note.* Mini-IPIP = Mini-International Personality Item Pool Scales; SF-12 = Short-Form Health Survey; DHLI = Digital Health Literacy Instrument. * = *p* < .05. ** = *p* < .01. *** = *p* < .001.
